# Technology overview and perspectives on next generation technologies

**DOI:** 10.1186/1753-6561-8-S4-O37

**Published:** 2014-10-01

**Authors:** Jaime Finguerut

**Affiliations:** 1 CTC - Centro de Tecnologia Canavieira S.A., Piracicaba, São Paulo, Brazil

## 

Today Brazil is the second world producer of fuel ethanol but has the best production system in terms of environmental impact and social positive impacts.

This is due to the use of sugarcane as feedstock that is perhaps the world champion crop in terms of biomass productivity and photosynthesis efficiency, even not being near to the theoretical limit.

In this presentation is shown how the sugarcane culture and its industrial processing co-evolved through adaptation to the production environment, in the agricultural area adaptation to the soil, climate and biotic and abiotic stresses and in the industrial area adaptation to the exposition to sugar prices volatility, to the very high interest rates that Brazil practiced for decades, together with economic uncertaints and high capital costs.

The present industrial process that is very efficient, very resilient and able to adjust the product mix in an ample range, is self- regulated in its ethanol production area, throughout forced adaptation of the yeast population, that evolve through the recycle in harsh and restrictive fermentation conditions.

This industrial process is very mature and for the last 10 years almost there was no significative gains because most of the possible gains with the present knowledge are there in operation in large scale. That means that new ideas are needed and that our Brazilian process in ready for new, revolutionary technologies such as the so called second generation technologies.

CTC is developing its own cellulosic ethanol production process learning from the evolution of the first generation. the idea is make possible using all carbohydrates produced by the sugarcane plant during its photosynthesis and not only the soluble sugars, sucrose mostly, as today. Since sucrose is only one third of the carbohydrates actually produced annually Brazil has the theorethical potential for tripling its output form the sugar mills.

Of course it will not be possible to have 100% efficiency but very significative gains will be possible as soon as new solutions for breaking the fiber of sugarcane are available for industrial use. That means that technical revolutions are needed e.g. to bring the enzymes costs close to presently practiced at the starch converting units and making possible to develop microrganisms that can efficiently ferment all sugars released by the enzymes including the pentoses such as xylose, that presently our fermentation workhorse Saccharomyces cerevisiae cannot ferment naturally. This fermentation process will have to be as efficient and productive as it is today in order to make possible producing the second generation ethanol as cheap or cheaper that it is today.

